# The Prognostic Role of Cortisol and Glucose Dynamics in Cardiogenic Shock-Insights from a Prospective Observational Cohort

**DOI:** 10.1007/s12265-025-10704-0

**Published:** 2025-10-18

**Authors:** Priyanka Boettger, Laura Pallmann, Jamschid Sedighi, Patrick Kellner, Henning Lemm, Roland Prondzinsky, Thomas Karrasch, Birgit Assmus, Karl Werdan, Michael Buerke

**Affiliations:** 1 Department of Internal Medicine I, Cardiology, Angiology and Intensive Care Medicine, Justus-Liebig-University Giessen, Klinikstrasse 33, 35392 Giessen, Hessen Germany; 2 Department of Medicine III, Cardiology, Angiology and Intensive Care Medicine, University Hospital Halle (Saale), Martin-Luther-University Halle-Wittenberg, Ernst-Grube-Strasse 40, 06120 Halle (Saale), Sachsen-Anhalt Germany; 3 Department of Anaesthesiology, Regio Kliniken, Agnes-Karll-Allee 17, 25337 Elmshorn, Schleswig-Holstein Germany; 4 Department of Internal Medicine II, Heart-Center Südwestfalen, Cardiology, Angiology and Intensive Care Medicine, St. Marien-Hospital, Kampenstrasse 51, 57072 Siegen, Nordrhein-Westfalen Germany; 5 Department of Internal Medicine III, Endocrinology, Justus-Liebig-University Giessen, Klinikstrasse 33, 35392 Giessen, Hessen Germany

**Keywords:** Cardiogenic shock, Glucose, Cortisol, Survivors, Predictors, Acute myocardial infarction, Stress response, Endocrine profiling, Translational research, Critical care, Biomarkers

## Abstract

Cardiogenic shock (CS) following myocardial infarction remains highly fatal. The prognostic value of dynamic metabolic markers—particularly glucose and cortisol—remains incompletely understood. In this prospective cohort study, 41 patients with infarction-related CS underwent serial blood sampling over 96 h. Plasma glucose and serum cortisol levels were measured repeatedly. Primary endpoint was in-hospital mortality. Admission glucose levels stratified as < 10, 10–15, and > 15 mmol/L were associated with rising mortality (36.4%, 43.8%, 50.0%; *p* = 0.47). Mortality was higher in patients without known diabetes. Early glucose normalization (≤ 6 h) correlated with improved survival (25% vs. 45%; *p* < 0.05). Cortisol levels were markedly elevated on admission. Survivors showed rapid decline; non-survivors had persistently high levels. Cumulative cortisol exposure (AUC₀–₉₆) was significantly lower in survivors (*p* = 0.016). Serial metabolic profiling identified early and sustained hyperglycaemia and hypercortisolaemia as independent predictors of mortality in infarction-related CS and potential targets for intervention.

**Graphical Abstract:**

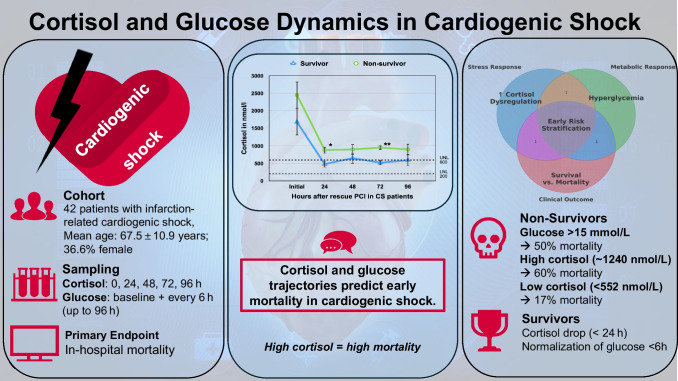

**Supplementary Information:**

The online version contains supplementary material available at 10.1007/s12265-025-10704-0.

## Introduction

Cardiogenic shock (CS) complicates up to 10% of acute coronary syndromes and remains associated with unacceptably high short-term mortality despite advances in early revascularization and mechanical circulatory support [13]. Current risk stratification relies mainly on hemodynamic and clinical parameters, yet reliable biomarkers that reflect the systemic stress response are lacking. Such markers could improve patient selection for advanced therapies and inform individualized treatment strategies.

Activation of the hypothalamic–pituitary–adrenal (HPA) axis and stress hyperglycemia represent hallmark features of the systemic stress response in critical illness [1] (Van den [24]). Cortisol supports vascular tone and catecholamine sensitivity but persistent hypercortisolemia may impair immune and metabolic regulation [3]. Similarly, stress-induced hyperglycemia results from gluconeogenesis, insulin resistance, and excess catabolic hormones, promoting endothelial dysfunction and adverse cardiovascular outcomes [14, 15]. Prior studies in acute myocardial infarction have shown that both overt diabetes and impaired fasting glucose predict adverse outcomes, including cardiogenic shock and mortality [27]. However, most investigations have relied on single baseline values, without capturing serial changes over the critical early course of shock, and rarely considered the interplay between cortisol and glucose as potentially synergistic drivers of adverse prognosis.

To address this knowledge gap, we conducted a prospective cohort study of patients with infarction-related cardiogenic shock, performing serial measurements of cortisol and glucose over the first 96 h of intensive care. We hypothesized that persistent elevations or insufficient decline of these biomarkers—and particularly their combined dysregulation—would be associated with increased mortality. By linking mechanistic endocrine and metabolic stress responses with patient outcomes, our study aims to provide translational insights that could inform future biomarker-guided strategies in cardiogenic shock.

## Material and Methods

A total of 45 consecutive patients with cardiogenic shock were prospectively enrolled in the Cardiogenic Shock Registry at the University Hospital. The diagnosis of cardiogenic shock was established during initial coronary angiography based on the following criteria: (1) systolic blood pressure < 90 mmHg, (2) clinical signs of systemic hypoperfusion, (3) indexed cardiac output < 2.2 L/min/m^2^, and (4) pulmonary capillary wedge pressure > 15 mmHg.

Patients referred for cardio-thoracic surgery during their hospital course were excluded, as subsequent surgical interventions would confound endocrine and metabolic measurements, limiting comparability with the non-surgical cohort. Patients with evidence of acute infection (bacterial, viral, or atypical) or glucocorticoid therapy at baseline or within the first 96 h were also excluded. After exclusions, the final study cohort comprised 41 patients. One predefined extreme cortisol outlier was excluded from cortisol trajectory/AUC analyses (cortisol set *n* = 40). No patient received systemic corticosteroids. All antidiabetic drugs were paused in state of shock. Clinical and laboratory data were collected on admission (“baseline”), immediately before and after percutaneous coronary intervention (PCI), six hours post-PCI, daily until day 7, and additionally on days 14 and 28. Routine blood sampling occurred at baseline, and at 24, 48, 72, and 96 h post-PCI. Blood glucose was assessed at baseline and subsequently every six hours until day 4, among those the first sample was fasting glucose. All patients were treated according to a standardized intravenous insulin infusion protocol. In line with current recommendations from the American Heart Association and the American Diabetes Association [9], insulin infusion was initiated when blood glucose exceeded 150 mg/dL (8.3 mmol/L), with a target range of 140–180 mg/dL (7.7–10 mmol/L) [11]. Capillary or venous glucose was monitored every hour in case of hyperglycemia (> 150 mg/dL) and insulin titration followed an institutional nurse-driven algorithm [23]. Subcutaneous insulin and non-insulin antidiabetic agents (e.g., DPP-4 inhibitors, SGLT2 inhibitors) were not used during shock. This uniform protocol ensured comparability across study participants.

At 5:00 a.m. each day, the central laboratory analyzed routine parameters including electrolytes, complete blood count, coagulation profile, cardiac biomarkers, renal and hepatic function tests, pancreatic enzymes, metabolic markers (e.g., lipids, proteins, glucose), and inflammatory markers. Additional plasma samples were centrifuged, aliquoted, and stored at − 80 °C for later analysis. Cortisol concentrations were determined from these samples in the certified Endocrinology Laboratory of the University Hospital.

### Statistic Methods

Statistical analyses were performed using IBM SPSS Statistics, version 27.0 (IBM Corp., Armonk, NY). Continuous variables were assessed for normality using the Shapiro–Wilk test and reported as mean ± standard error (SE) or median with interquartile range, as appropriate. Group comparisons used t-tests or Mann–Whitney U tests for continuous data, and Chi-square or Fisher’s exact tests for categorical data. Statistical significance was defined as *p* < 0.05 (two-sided). Temporal changes in continuous variables were evaluated using repeated-measures ANOVA or linear mixed-effects models with Bonferroni correction. Area under the curve (AUC) from baseline to 72 h was calculated using the trapezoidal rule and compared using t-tests. Correlations were assessed with Pearson’s or Spearman’s coefficients, and effect sizes reported as Cohen’s d.

Visualizations were created using SPSS and Apple Numbers, with error bars reflecting standard error unless stated otherwise.

## Results

After the predefined exclusions, a final cohort of 41 patients with infarction-related cardiogenic shock was prospectively enrolled in the Cardiogenic Shock Registry at our academic hospital. In all cases, cardiogenic shock occurred secondary to acute myocardial infarction (STEMI or NSTE-ACS) Three patients were excluded from final analysis due to referral for coronary artery bypass grafting (CABG) and incomplete sampling, yielding a final cohort of 41 patients.

The cohort comprised 15 women (36.6%) and 26 men (63.4%). The overall in-hospital mortality rate was 43.9% (95% CI, 28.3–59.4%), consistent with contemporary cardiogenic shock registries. Among women, 6 of 15 (40.0%; 95% CI, 16.3–67.6%) died, compared with 12 of 26 (46.2%; 95% CI, 26.6–66.6%) men. This difference was not statistically significant (*p* = 0.70, Fisher’s exact test). Survivors and non-survivors did not differ significantly by sex distribution (*p* = 0.70).

Baseline demographic and clinical parameters—including age, cardiovascular risk factors, comorbidities, and BMI—are summarized in Table 1, with subgroup comparisons by sex and survival status. The patient population reflects the typical clinical spectrum of infarction-related cardiogenic shock and appears broadly representative of other real-world registry cohorts.

**Table 1:** Baseline characteristics of the study population according to survival status, sex, and age group

Variable	All Patients (*n* = 41)	Survivors (*n* = 23)	Non-survivors (*n* = 18)	Male (*n* = 26)	Female (*n *= 15)	Age < 70 y (*n* = 18)	Age ≥ 70 y (*n* = 23)
Age, y	67.5 ± 10.9	64.9 ± 9.8	70.8 ± 11.4	66.2 ± 9.6	69.7 ± 10.1	59.2 ± 8.5	76.2 ± 9.7
Weight, kg	87.1 ± 13.6	88.7 ± 12.4	84.9 ± 15.2	84.1 ± 14.1	88.8 ± 12.2	91.4 ± 11.7	82.6 ± 14.6
Height, cm	171.2 ± 8.2	174.3 ± 7.4	167.3 ± 8.6	176.0 ± 6.8	162.9 ± 7.2	177.0 ± 6.3	165.2 ± 7.8
BMI, kg/m^2^	29.9 ± 6.3	29.3 ± 6.1	30.4 ± 6.6	28.7 ± 5.9	31.5 ± 6.7	29.4 ± 5.8	30.2 ± 6.5
Prior MI, *n* (%)	10 (24%)	7 (30%)	3 (17%)	3 (12)	7 (47%)	4 (22%)	6 (26%)
Prior HF, *n* (%)	17 (41%)	9 (39%)	8 (44%)	5 (19)	12 (80%)	5 (28%)	12 (52%)
Hypertension, *n* (%)	26 (63%)	17 (74%)	9 (50%)	10 (38%)	15 (100%)*	13 (72%)	13 (57%)
Type 2 DM, *n* (%)	24 (59%)	14 (61%)	10 (56%)	12 (46%)	12 (80%)	10 (56%)	14 (61%)
Dyslipidemia, *n* (%)	8 (20%)	6 (26%)	2 (11%)	2 (8%)	6 (40%)	5 (28%)	3 (13%)
Current smoker, *n* (%)	9 (22%)	6 (26%)	3 (17%)	2 (8%)	7 (47%)	7 (39%)	2 (9%)

Values are presented as mean ± SD or number (%). *Y* years, *kg*  kilograms, *BMI*  body mass index, *HPT* hypertension, *DM* diabetes mellitus, *HLP* hyperlipidemia

### Demographics and Prehospital Clinical Status

The mean age at hospital admission was 67.5 ± 10.9 years. Women were on average older than men (69.7 ± 10.1 vs. 66.2 ± 9.6 years; *p* = 0.18). Survivors were significantly younger than non-survivors (64.9 ± 9.8 vs. 70.8 ± 11.4 years; *p* = 0.048), identifying age as a potential prognostic factor. The cohort had a mean body weight of 87.1 ± 13.6 kg (range: 60–125 kg) and mean height of 171.2 ± 8.2 cm (range: 145–190 cm), yielding an average BMI of 29.9 ± 6.3 kg/m^2^. Women had significantly higher BMI values than men (31.5 ± 6.7 vs. 28.7 ± 5.9 kg/m^2^; *p* = 0.046); 60% of women and 30% of men were obese (BMI > 30 kg/m^2^). However, BMI was not significantly associated with in-hospital mortality (*r *= 0.11, *p* = 0.51).

Prehospital clinical status reflected the severity of illness: 78% (32/41) of patients were intubated on arrival, and 34% (14/41) had undergone prehospital cardiopulmonary resuscitation (CPR). Among these, 6 (43%) presented with ventricular fibrillation. A total of 16 patients (39%) received revascularization therapy before hospital arrival: 11 received thrombolysis, 4 underwent percutaneous transluminal coronary angioplasty (PTCA), and 1 received both. In contrast, 7 patients (17%) had no prehospital reperfusion initiated before admission.

### Prehospital Cardiac Arrest Rhythms

Out-of-hospital cardiac arrest (OHCA) was documented in 14 of 41 patients (34%) with infarction-related cardiogenic shock. The initial rhythms comprised ventricular fibrillation (VF) in 6 patients (43%), pulseless electrical activity (PEA) in 5 (36%), and asystole in 3 (21%).

When stratified by survival, the distribution of rhythms was broadly comparable between groups. Among survivors (*n* = 23), 5 patients (22%) had experienced OHCA (3 VF, 1 PEA, 1 asystole), whereas among non-survivors (*n* = 18), 9 patients (50%) presented with OHCA (3 VF, 4 PEA, 2 asystole). Although there was a numerical trend toward more non-shockable rhythms (PEA/asystole) in non-survivors (33% vs. 9% in survivors), this difference did not reach statistical significance (*p* = 0.18, Fisher’s exact test).

These findings suggest that while the type of prehospital arrest rhythm may influence prognosis, the groups in this cohort were relatively balanced with respect to OHCA and initial rhythm. Thus, differences in cortisol and glucose dynamics between survivors and non-survivors are unlikely to be confounded by rhythm distribution.

### Circulatory Parameters and Hemodynamic Support

At admission, the mean systolic blood pressure (SBP) of the cohort was 104 mmHg (95% CI, 97.5–110.5), and the mean heart rate was 94 beats/min (95% CI, 89–99). Survivors presented with higher SBP compared to non-survivors (116.6 ± 12.4 vs. 88.5 ± 11.3 mmHg; *p* = 0.001), while heart rate did not differ. SBP values converged during the first 24 h (108.1 vs. 100.1 mmHg, *p* = 0.06). The mean cardiac index improved from 1.96 ± 0.2 L/min/m^2^ at baseline to 2.57 ± 0.3 L/min/m^2^ by day one, reflecting successful revascularization and stabilization. Serum troponin I was higher in non-survivors (26.1 ± 9.1 µg/L) than survivors (20.8 ± 5.8 µg/L), though not statistically significant (*p* = 0.31).

Most patients required vasoactive support. Norepinephrine was administered in 83% (median peak 0.32 µg/kg/min, IQR 0.21–0.48), dobutamine in 80% (median 7.5 µg/kg/min, IQR 5.0–10.0), and epinephrine in 22% (median 0.12 µg/kg/min, IQR 0.08–0.19). Use and peak doses did not differ significantly between survivors and non-survivors. Cumulative vasopressor exposure over 48 h was also similar across groups. Mechanical circulatory support (MCS) was required in 76% of patients (IABP, *n* = 24; Impella, *n* = 5; VA-ECMO, *n* = 2), with comparable use between survivors and non-survivors (74% vs. 78%, *p* = 0.81).

In multivariable analysis adjusting for norepinephrine dose and MCS use, cumulative cortisol exposure (AUC₀–₉₆) remained independently associated with in-hospital mortality (OR 1.42 per 10,000 nmol·h/L increase, 95% CI 1.05–1.92; *p* = 0.02), whereas admission glucose was not (OR 1.08 per 1 mmol/L, 95% CI 0.94–1.23; *p* = 0.27). This indicates that the prognostic role of cortisol was not confounded by hemodynamic support intensity.

### Severity Scores and their Association with Cortisol and Glucose

At admission, median severity scores were APACHE II 24 (IQR 20–29), APACHE III 74 (IQR 63–92), and SOFA 10 (IQR 8–12), consistent with a critically ill shock population. Non-survivors presented with significantly higher scores compared with survivors (APACHE II: 28 vs. 22, *p* = 0.006; APACHE III: 88 vs. 66, *p* = 0.003; SOFA: 12 vs. 8, *p *< 0.001), and these differences persisted throughout the first 96 h. SOFA declined steadily in survivors (8 → 3), but remained stable or increased in non-survivors (12 → 14; group × time interaction *p* < 0.001).

Cortisol concentrations correlated positively with severity. Admission cortisol showed moderate associations with SOFA (*r* = 0.42, *p* = 0.01) and APACHE II (*r* = 0.39, *p* = 0.02), and cumulative cortisol exposure (AUC₀–₉₆) remained higher in patients with persistently elevated scores. By contrast, glucose was only weakly associated with severity indices (SOFA: *r* = 0.18, *p* = 0.27; APACHE II: *r* = 0.15, *p* = 0.32), and these correlations did not reach statistical significance.

Taken together, these findings indicate that cortisol not only reflects the extent of organ dysfunction but also retains independent prognostic value beyond established severity scores, while glucose appears more as a parallel stress marker without independent predictive strength (see Supplement Fig. 1).

### Cortisol Dynamics

**Fig. 1:**
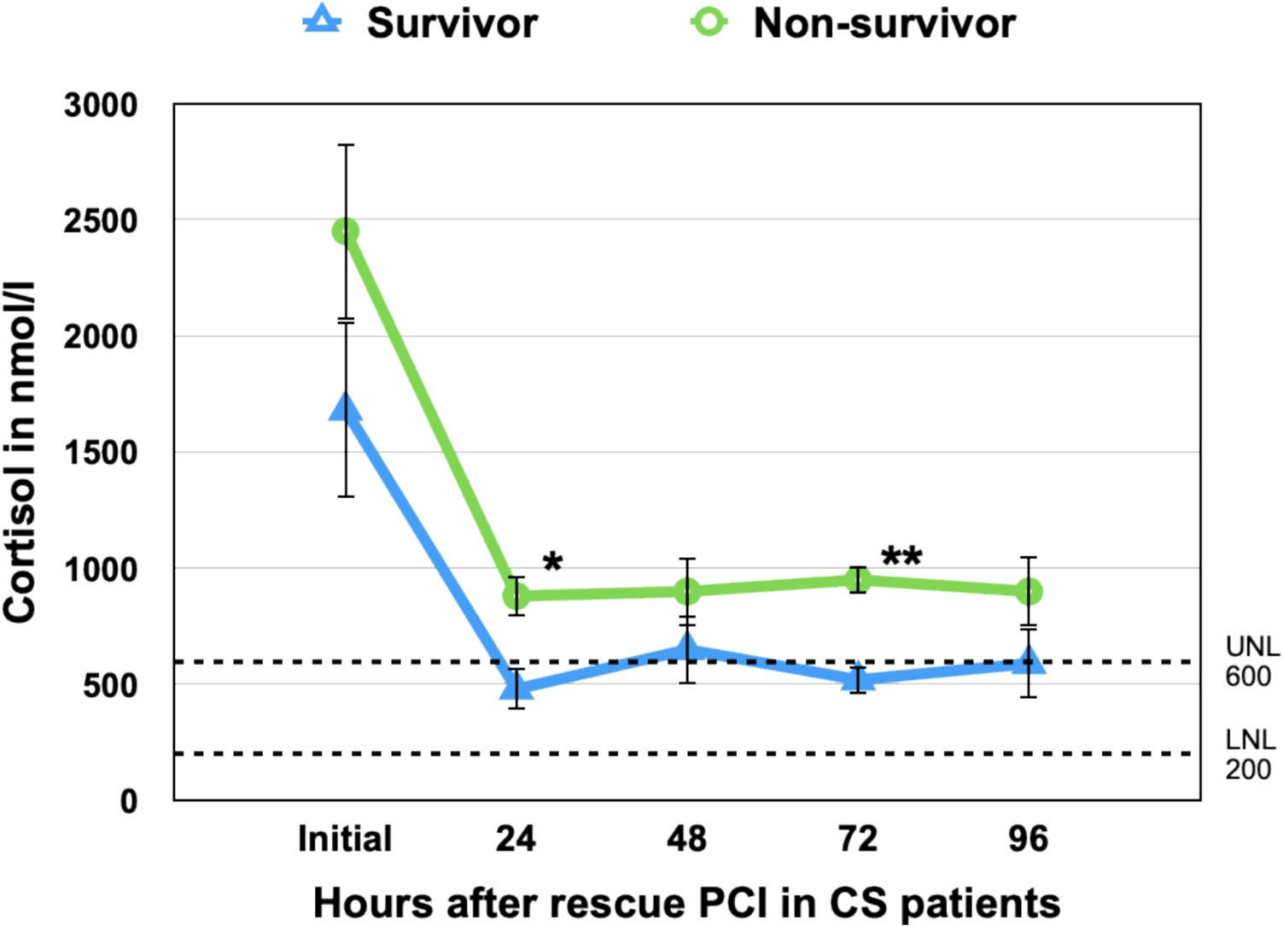
Temporal Changes in Serum Cortisol Levels in Survivors and Non-Survivors With Infarction-Related Cardiogenic Shock Mean serum cortisol concentrations (± standard error) are shown at admission and at 24, 48, 72, and 96 h post-admission, stratified by in-hospital survival status. Survivors (blue triangles, dashed line) exhibited a rapid decline in cortisol levels, reaching near-normal range within 24 h (389.8 nmol/L). In contrast, non-survivors (green circles, solid line) showed persistently elevated cortisol throughout the observation period, with levels at 24 and 48 h remaining significantly higher (*p* = 0.003 and *p* = 0.020, respectively). The difference in cumulative cortisol exposure (AUC₀–₉₆) between groups was also significant (*p* = 0.016), underscoring persistent hypercortisolemia as a marker of poor prognosis

At the time of admission, before coronary intervention, the mean serum cortisol concentration was markedly elevated at 2316.9 ± 495.7 nmol/L (reference range: 180–630 nmol/L), with a median of 1068.5 nmol/L. The range extended from 119 to 16,212 nmol/L; the latter value was excluded from analysis as a statistical outlier. Following this exclusion, 40 patients were included in the cortisol trajectory analysis. Cortisol concentrations declined significantly over the first 24 h from 1919.9 ± 428.7 nmol/L to 599.1 ± 125.4 nmol/L (*p* = 0.0006), returning to the normal range in most patients. Survivors consistently exhibited lower cortisol concentrations than non-survivors across all timepoints. At 24 h, mean cortisol in survivors had normalized to 389.8 nmol/L, whereas in non-survivors it remained elevated at 729.7 nmol/L. Although the initial difference at admission was not statistically significant (*p* = 0.29), between-group differences became significant at 24 h (*p* = 0.003) and remained so at 72 h (*p* = 0.020), suggesting that persistent hypercortisolemia is associated with worse outcomes (see Fig. 1).

### Cortisol Exposure Over Time (AUC Analysis)

To quantify cumulative cortisol exposure, we calculated the area under the curve (AUC₀–₉₆) from admission to 96 h using the trapezoidal rule, based on group mean cortisol values.

**Fig. 2:**
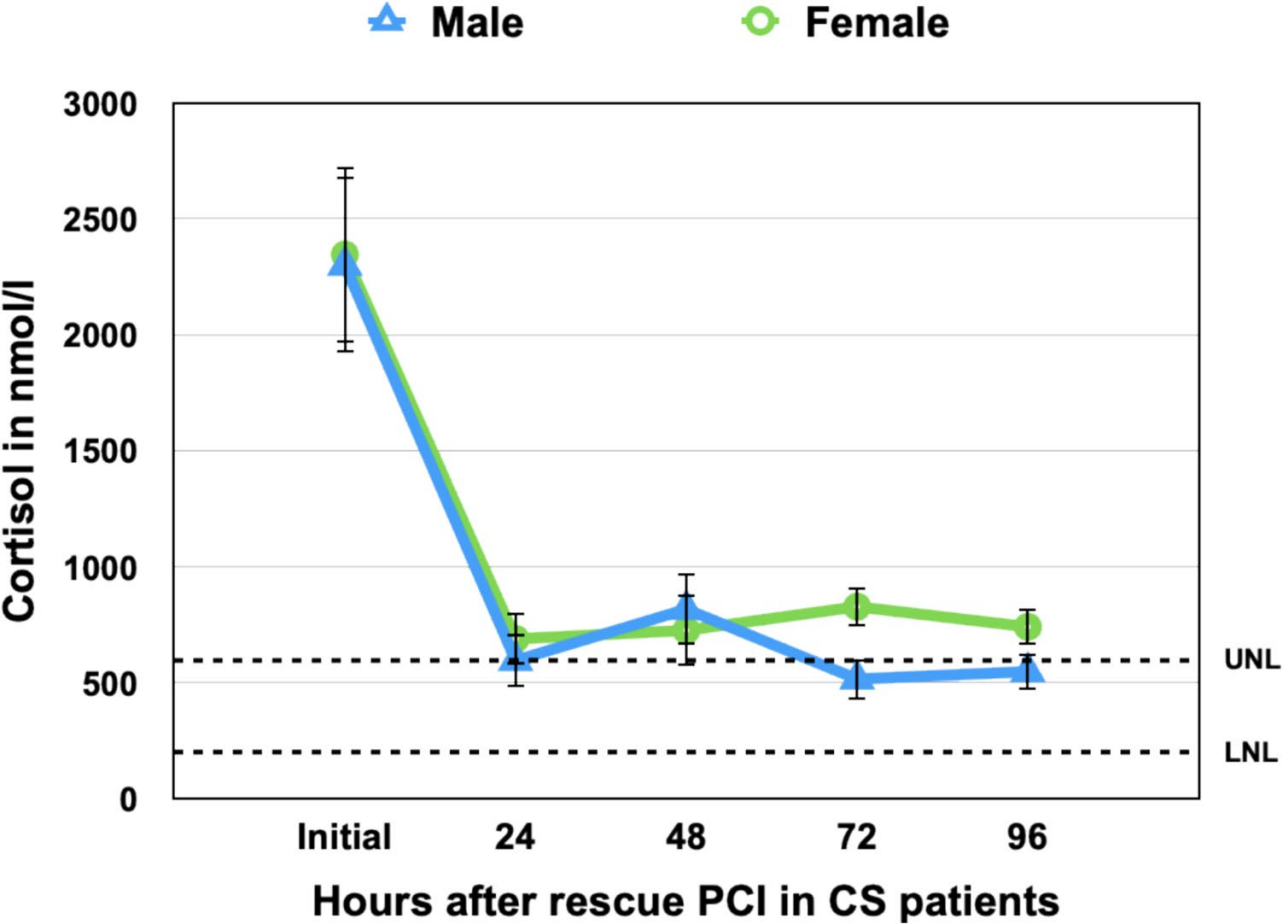
Sex-Specific Cortisol Kinetics Following Rescue PCI in Cardiogenic Shock Mean serum cortisol concentrations (± standard error) are shown at admission and through 96 h post–rescue PCI, stratified by sex: males (blue triangles, solid line) and females (green circles, solid line). Both sexes demonstrated a steep decline in cortisol levels during the first 24 h, from supraphysiologic concentrations to near the upper limit of the normal range (UNL = 630 nmol/L, dashed line). No statistically significant differences were observed between males and females at any timepoint (*p* > 0.05 for all comparisons), and cumulative cortisol exposure over 96 h (AUC₀–₉₆) was comparable (*p* = 0.38), suggesting cortisol kinetics were independent of sex in this cohort

**Fig. 3:**
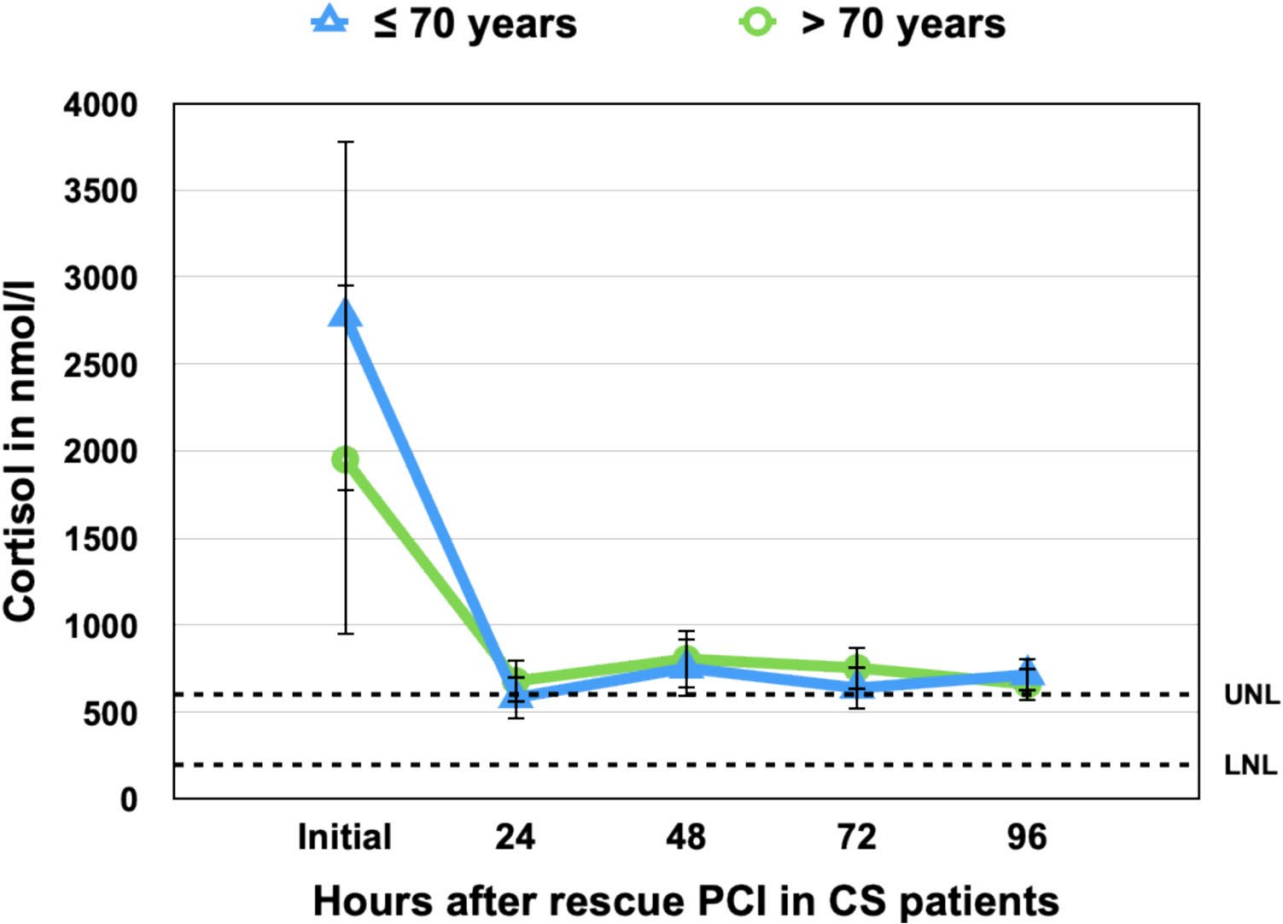
Cortisol Kinetics Following Rescue PCI in Cardiogenic Shock, Stratified by Age Group Mean serum cortisol concentrations (± standard error) are shown at admission and up to 96 h post–rescue PCI, stratified by age ≤ 70 years (blue triangles) and > 70 years (green circles). Both age groups exhibited a sharp decline in cortisol levels within the first 24 h. Although patients ≤ 70 years had higher initial cortisol levels, this difference did not persist beyond the first day. Cortisol levels stabilized near the upper limit of the normal range (UNL = 630 nmol/L, dashed line) across timepoints. No statistically significant differences were observed between age groups at individual timepoints (*p* > 0.05 for all comparisons). LNL = lower normal limit (180 nmol/L)

Female patients exhibited no significant differences in total cortisol exposure compared to male patients (AUC₀–₉₆: 90,982 vs. 81,757 nmol·h/L (*p* = 0.38), and no significant differences were detected at any individual time point (Fig. 2). In contrast, non-survivors showed substantially greater cumulative cortisol levels than survivors (AUC₀–₉₆: 97,988 vs. 65,476 nmol·h/L; *p* = 0.016), supporting the association between persistent hypercortisolemia and adverse outcomes in infarction-related cardiogenic shock (Fig. 1). The difference in AUC between survival groups was more pronounced than at any single timepoint, highlighting the value of temporal hormone profiling over isolated measurements. When stratified by age, cumulative cortisol exposure (AUC₀–₉₆) was similar in patients ≤70 and >70 years, indicating that age did not modify total cortisol burden during the first 96 hours (Fig. 3).

These findings suggest that dynamic cortisol suppression within the first 24–48 h may serve as a prognostic marker, while sex-related differences in cortisol kinetics appear less clinically relevant.

### Risk Stratification by Cortisol Thresholds

To assess the prognostic relevance of admission cortisol concentrations, patients were stratified into three groups based on thresholds previously validated in septic shock [19]: low (< 552 nmol/L; *n* = 6), intermediate (552–1240 nmol/L; *n* = 18), and high (> 1240 nmol/L; *n* = 15). In-hospital mortality increased stepwise across these strata: 16.7% in the low group (1/6), 38.9% in the intermediate group (7/18), and 60.0% in the high group (9/15). This trend reached statistical significance (*p* = 0.047, Chi-square test for trend).

Although confidence intervals were wide due to small sample sizes, the odds of death were markedly elevated in the high cortisol group compared to the low group (OR = 7.50; 95% CI: 0.70–79.7) and moderately elevated compared to the intermediate group (OR = 2.40; 95% CI: 0.65–9.01). A positive correlation between cortisol category and in-hospital mortality was observed (Spearman’s *r* = 0.42, *p* = 0.008), suggesting a dose–response relationship between stress hypercortisolemia and adverse outcome.

**Fig. 4:**
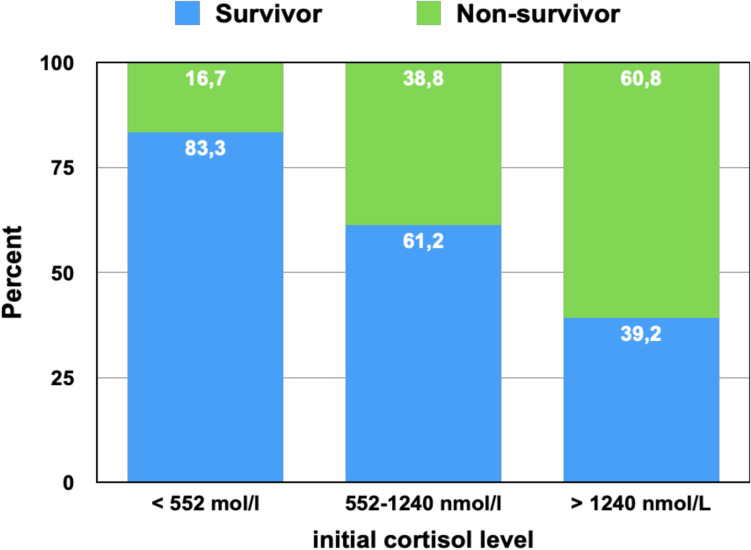
In-hospital mortality by admission cortisol levels in infarction-related cardiogenic shock. Stacked bar chart depicting the proportion of survivors (blue) and non-survivors (green) stratified by admission serum cortisol concentration: Low (< 552 nmol/L, ***n* = **6), Intermediate (552–1240 nmol/L, n = 18), High (> 1240 nmol/L, n = 15) A significant stepwise increase in in-hospital mortality was observed with rising cortisol levels: 16.7% in the low group, 38.9% in the intermediate group, and 60.0% in the high group (***p* = **0.047, Chi-square test for trend). The odds of death in the high cortisol group were 7.5 times higher than in the low group (OR = 7.50, 95% CI: 0.70–79.7), supporting a dose–response relationship between admission hypercortisolemia and adverse outcomes. These findings underscore the prognostic relevance of early endocrine profiling in infarction-related cardiogenic shock.

These findings support the clinical utility of cortisol-based risk stratification at admission. As illustrated in Fig. 4, baseline cortisol levels inversely correlated with survival. Importantly, subgroup analyses by sex and age (< 70 vs. ≥ 70 years) revealed no significant differences in cortisol kinetics (Table 4), indicating that the prognostic impact of cortisol was independent of demographic characteristics.

### Diabetes Mellitus and Blood Glucose

At the time of admission, the mean blood glucose level in the cohort was 12.9 ± 1.2 mmol/L (median: 12.4 mmol/L; range: 4.7–40.2 mmol/L), significantly exceeding the upper limit of normal (< 6.1 mmol/L). Only 4 of 41 patients (9.8%) presented with glucose levels within the normal range. The prevalence of diagnosed type 2 diabetes mellitus (T2DM) was high, with 24 of 41 patients (58.5%) previously diagnosed, and an equal sex distribution (12 males, 12 females). In-hospital mortality did not differ significantly between patients with and without diabetes. Among those with T2DM, 10 of 24 (41.7%) died, compared to 8 of 17 (47.1%) without T2DM (*p* = 0.72, Fisher’s exact test), suggesting that baseline diabetes status was not independently associated with mortality in this cohort. Insulin therapy was initiated in 37 of 41 patients (90.2%) during hospitalization. The 4 patients who did not receive insulin included two who died within the first 24 h—likely prior to initiation of glycemic control—and two who maintained stable glucose levels below 8.8 mmol/L throughout admission.

**Fig. 5:**
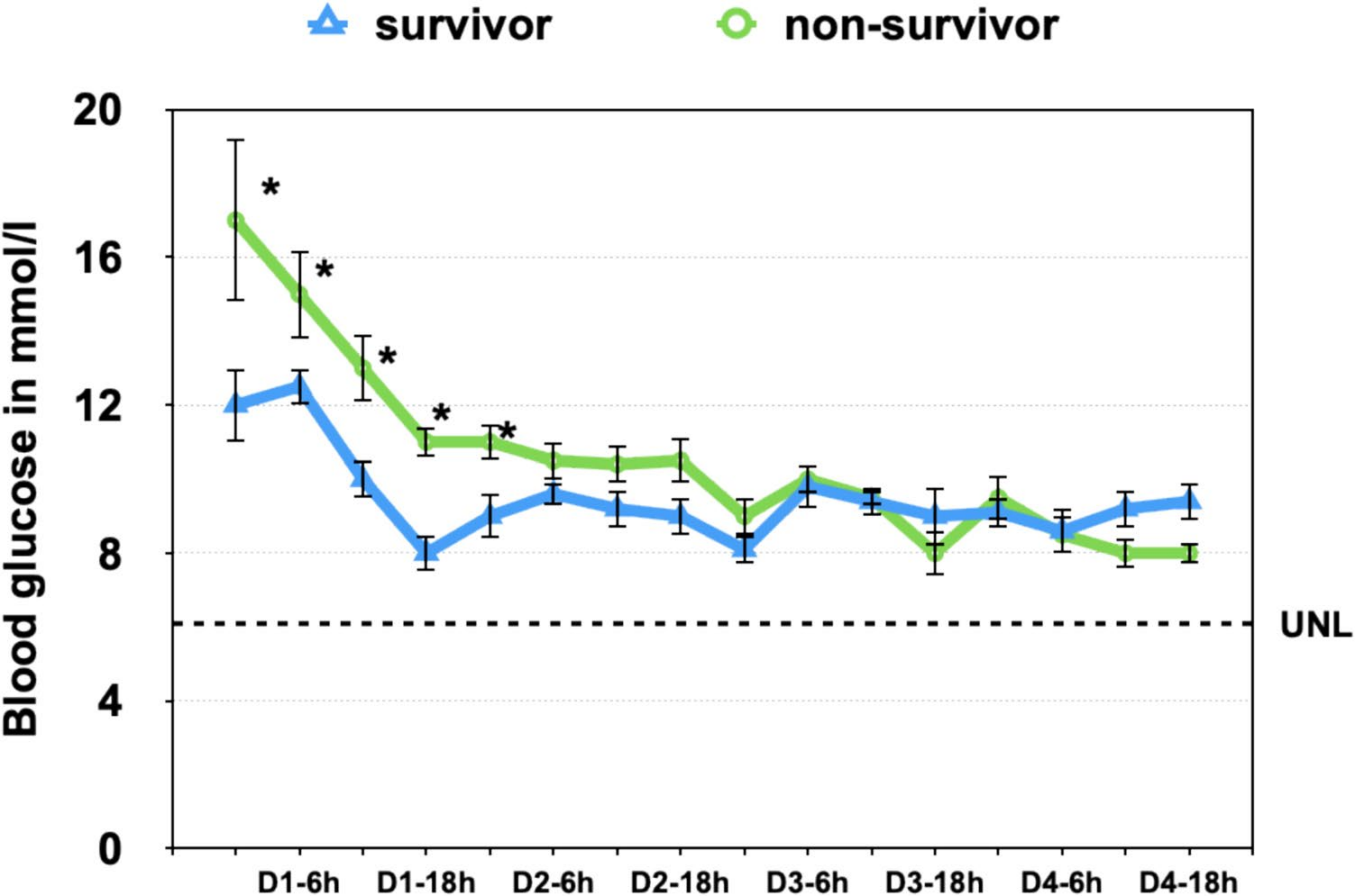
Temporal Blood Glucose Profiles in Survivors and Non-Survivors with Cardiogenic Shock Mean blood glucose concentrations (± standard error) are shown over 96 h in survivors (blue triangles) and non-survivors (green circles) following admission for infarction-related cardiogenic shock. Both groups exhibited an initial decline in glucose levels, with non-survivors starting from significantly higher values at admission. Despite similar glycemic trajectories thereafter, glucose levels remained moderately elevated in non-survivors throughout the observation period. However, no statistically significant differences were observed at individual timepoints (*p* > 0.05 across all comparisons). These findings suggest that the extent of initial hyperglycemia may carry more prognostic weight than subsequent glycemic fluctuations

Over the first 96 h, blood glucose levels showed a general downward trend under insulin treatment, with transient increases in the early morning and midday periods, consistent with diurnal stress responses. Time-course analysis by survival status revealed similar glycemic trajectories in both groups, with no statistically significant differences at any timepoint (*p* > 0.05 across all measurements; see Fig. 5). Among a total of 500 glucose measurements, the lowest value recorded was 3.4 mmol/L, and values < 5.0 mmol/L were observed at 9 distinct timepoints (1.8%), suggesting that hypoglycemia was rare and transient. Glucose levels declined markedly over the first four days after admission in both sexes and age groups. Temporal glucose profiles showed no consistent differences between male and female patients (Table 2) or between patients younger and older than 70 years (Table 3). Repeated-measures analysis confirmed a significant main effect of time (p 0.05). By contrast, cortisol concentrations stratified by sex and age are shown in Table 4.

**Table 2:** Temporal profile of blood glucose levels (mmol/L) by sex during the first four days post-admission

	Male	Female
*initial Glucose*	13,10 ± 1,45	17,33 ± 3,24
*D 1 Glucose 6 h*	10,83 ± 0,97	17,36 ± 2,30
*D 1 Glucose 12 h*	10,03 ± 1,06	13,09 ± 0,74
*D 1 Glucose 18 h*	8,56 ± 1,09	9,72 ± 0,75
*D 2 Glucose 0 h*	9,15 ± 0,58	9,33 ± 0,71
*D 2 Glucose 6 h*	9,40 ± 0,59	10,38 ± 0,64
*D 2 Glucose 12 h*	8,44 ± 0,45	10,34 ± 0,86
*D 2 Glucose 18 h*	8,41 ± 0,33	8,61 ± 0,63
*D 3 Glucose 0 h*	8,56 ± 0,51	11,37 ± 1,51
*D 3 Glucose 6 h*	8,63 ± 0,39	10,44 ± 0,99
*D 3 Glucose 12 h*	8,67 ± 0,44	8,73 ± 0,87
*D 3 Glucose 18 h*	8,14 ± 0,41	8,48 ± 0,56
*D 4 Glucose 0 h*	8,38 ± 0,66	8,24 ± 0,51

Mean values (± standard error) of glucose levels in mmol/L, stratified by sex; *D *Day

**Table 3:** Temporal profile of blood glucose Levels (mmol/L) by age group during the first Four days post-admission

	< 70 years	≥ 70 years
*initial Glucose*	16,17 ± 4,51	14,32 ± 1,43
*D 1 Glucose 6 h*	12,89 ± 2,40	13,95 ± 1,27
*D 1 Glucose 12 h*	9,89 ± 1,17	12,46 ± 0,87
*D 1 Glucose 18 h*	6,66 ± 0,64	11,12 ± 1,05
*D 2 Glucose 0 h*	8.08 ± 0,40	10,18 ± 0,67
*D 2 Glucose 6 h*	8,97 ± 0,58	10,44 ± 0,61
*D 2 Glucose 12 h*	8,77 ± 0,59	9,49 ± 0,67
*D 2 Glucose 18 h*	7,79 ± 0,34	9,12 ± 0,47
*D 3 Glucose 0 h*	9,83 ± 1,45	9,61 ± 0,63
*D 3 Glucose 6 h*	9,87 ± 1,04	9,04 ± 0,39
*D 3 Glucose 12 h*	9,57 ± 0,80	8,11 ± 0,48
*D 3 Glucose 18 h*	8,79 ± 0,47	7,91 ± 0,45
*D 4 Glucose 0 h*	8,69 ± 0,39	7,51 ± 0,53

Mean values (± standard error) of blood glucose levels in mmol/L, stratified by age < 70 years and ≥ 70 years, *D* Day

**Table 4:** Temporal Profile of Serum Cortisol Concentrations (nmol/L) by Sex and Age Group

	Initial	24 h	48 h	72 h	96 h
*female*	2345,00 ± 520,59	690,39 ± 111,61	727,00 ± 86,98	829,83 ± 128,44	742,42 ± 114,16
*male*	2299,00 ± 750,56	597,48 ± 114,05	818 ± 156,87	517,00 ± 87,26	649,1 ± 78,88
< *70 years*	2776,63 ± 1010,25	583,11 ± 128,58	753,44 ± 171,56	638,36 ± 124,12	715,71 ± 97,65
≥ *70 years*	1949,10 ± 392,59	678,94 ± 106,26	806,05 ± 117,17	654,00 ± 100,75	660,81 ± 89,66

Mean values (± standard error) of cortisol concentrations in nmol/L, stratified by sex and age < 70 vs. ≥ 70 years over the first 96 h of hospitalization

### Admission Hyperglycemia and Risk Stratification

To further evaluate the prognostic value of early hyperglycemia, the cohort was stratified into three subgroups based on admission glucose levels, independent of pre-existing diabetes mellitus:

- Group 1: < 10 mmol/L (*n* = 11)
- Group 2: 10–15 mmol/L (*n* = 16)
- Group 3: > 15 mmol/L (*n* = 14)

**Fig. 6:**
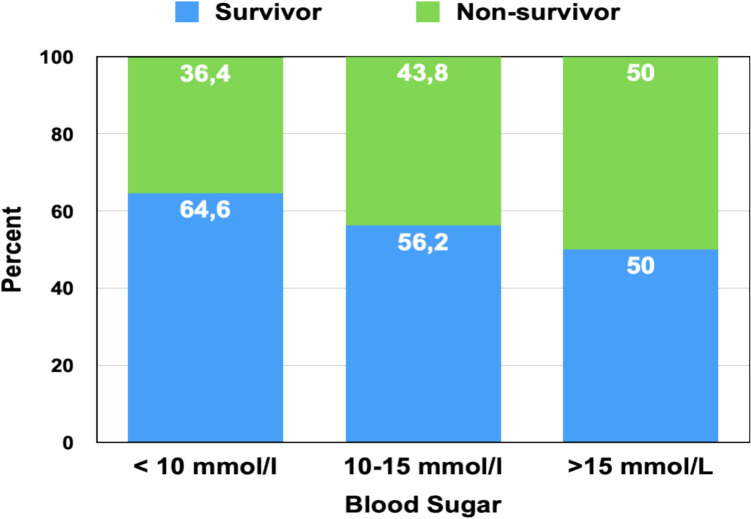
In-Hospital Mortality by Admission Glucose Range in Cardiogenic Shock Patients Stacked bar chart showing proportions of survivors (blue) and non-survivors (green) according to admission blood glucose levels: < 10 mmol/L (*n* = 11), 10–15 mmol/L (*n* = 16), and > 15 mmol/L (*n* = 14). A trend toward increased mortality was observed with rising glucose levels: survival decreased from 64.6% in the < 10 mmol/L group to 50.0% in the > 15 mmol/L group. While the differences were not statistically significant (*p*** = **0.19), the findings support the clinical relevance of admission hyperglycemia as a potential risk marker in infarction-related cardiogenic shock

A numerically progressive increase in mortality was observed with rising glucose strata (Fig. 6). In-hospital mortality was 36.4% (4/11) in Group 1, 43.8% (7/16) in Group 2, and 50.0% (7/14) in Group 3. Although this did not reach statistical significance (*p* = 0.47, Chi-square test), the directionality was consistent with prior analyses and suggests a clinically meaningful relationship [13].

The absolute risk difference between the lowest and highest glucose groups was 13.6% (95% CI: − 25.7 to + 51.5, *p* = 0.70, Fisher’s exact test). A binary comparison of mortality between patients with admission glucose > 15 mmol/L versus ≤ 15 mmol/L revealed a numerically higher mortality in the hyperglycemic group (50.0% vs. 40.7%, risk difference 9.3%; 95% CI: − 19.8 to + 38.5; *p* = 0.55, Fisher’s exact test), which was not statistically significant.

**Fig. 7:**
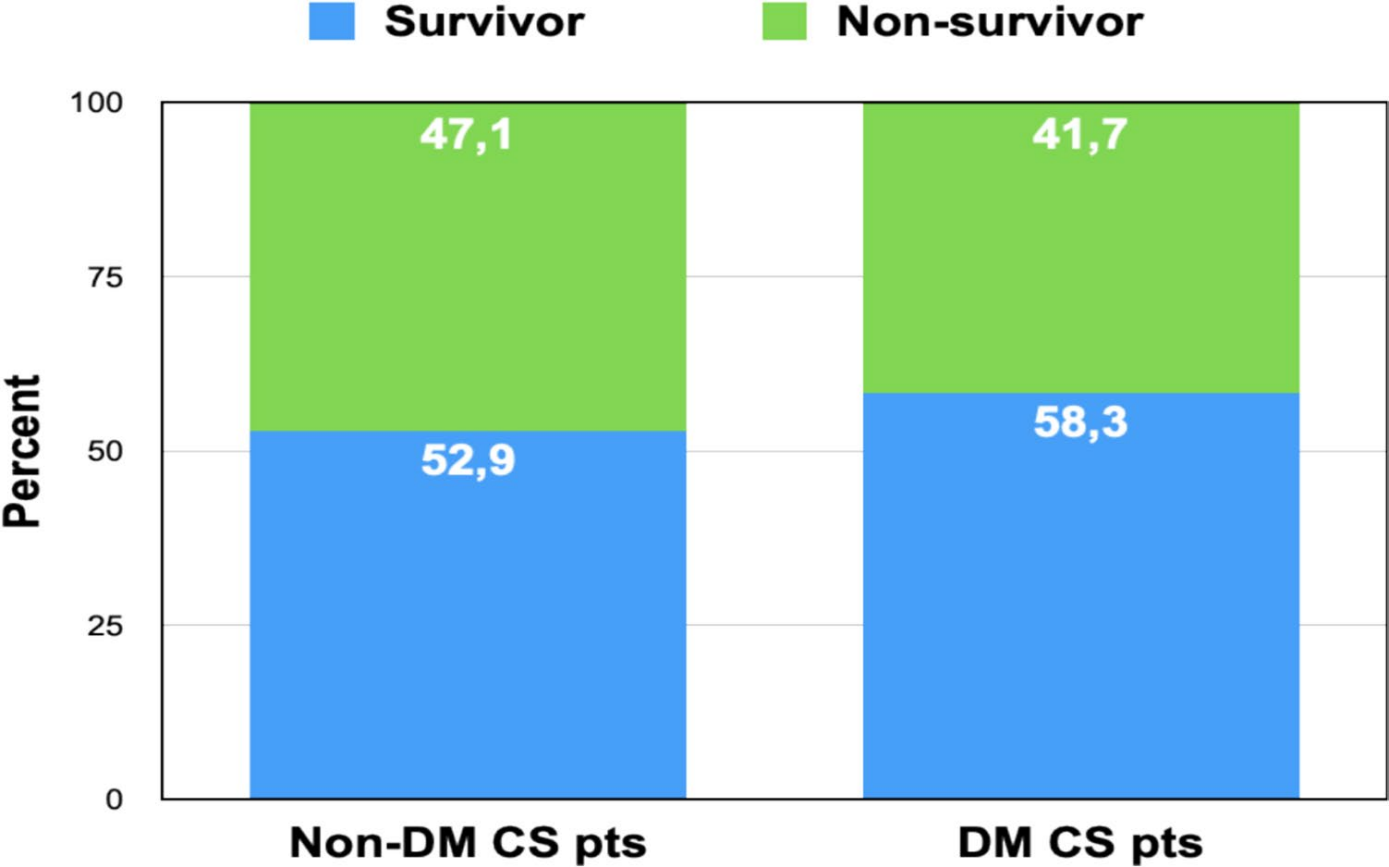
In-Hospital Mortality in Patients With and Without Type 2 Diabetes Mellitus Stacked bar chart displaying survival (blue) and mortality (green) proportions in patients with known type 2 diabetes mellitus (T2DM) versus those without diabetes at admission. Among patients with T2DM (*n* = 24), 58.3% survived and 41.7% died. In patients without T2DM (*n*** = **17), the survival rate was 52.9%, with 47.1% mortality. The difference in outcome between groups was not statistically significant (p = 0.72, Fisher’s exact test), suggesting that pre-existing diabetes was not an independent predictor of in-hospital mortality in this cohort of infarction-related cardiogenic shock patients

We also examined mortality by diabetes status. Among patients with diagnosed type 2 diabetes mellitus (T2DM, *n* = 24), mortality was 41.7%, compared to 47.1% in patients without T2DM (*n* = 17) (Fig. 7), which was not statistically significant (*p* = 0.72, Fisher’s exact test). These counterintuitive findings may be explained by pre-existing antihyperglycemic therapy, resulting in more attenuated glucose levels at admission. Mean glucose on admission in patients with T2DM was numerically lower than in those without T2DM (12.3 ± 3.8 vs. 13.6 ± 4.1 mmol/L), though this difference did not reach statistical significance (*p* = 0.26, unpaired *t*-test).

### Cumulative Glucose Exposure (AUC Analysis)

Cumulative glucose exposure over 96 h (AUC₀–₉₆) was calculated using the trapezoidal rule. The average AUC was slightly higher in non-survivors (1,198 mmol·h/L) compared to survivors (1,084 mmol·h/L), although this difference did not reach statistical significance (*p* = 0.11). Glucose trajectories remained similar across groups, but the extent and persistence of early hyperglycemia appear to influence outcome. However, mortality was lower in CS patients when admission glucose levels as well as serial levels within the first 24 h were lower. These results support the relevance of early glucose control and reinforce admission glucose as a simple, modifiable predictor of mortality in this setting.

## Discussion

Cardiogenic shock (CS) following myocardial infarction initiates a profound neuroendocrine response, yet the prognostic significance of hormonal stress markers remains underexplored. In this prospective cohort, we demonstrate that patients with infarction-related CS exhibit marked hypercortisolemia at presentation, with mean cortisol levels (2316.9 ± 482.1 nmol/L) nearly fivefold above the upper reference limit. These levels far exceed those reported in septic shock cohorts, including the Cohort of Annane et al. [1] (938 nmol/L), Ray et al. [18] (1532 nmol/L), and Bendel et al. [2] (793 nmol/L). In comparison, Ho et al. observed basal cortisol concentrations of 880± 79 nmol/L in septic shock, 417 ± 45 nmol/L in sepsis, and 352 ± 34 nmol/L in healthy controls [12]. This exaggerated adrenal response likely reflects the combined physiological insult of myocardial necrosis and systemic hypoperfusion.

Importantly, cortisol dynamics differed by outcome: survivors showed a more rapid decline, with levels normalizing within 24 h, whereas non-survivors exhibited persistent hypercortisolemia. Stratification of patients by admission cortisol revealed a stepwise increase in mortality, in line with prior findings by Sam et al. in septic shock [19]. Similar U-shaped associations between cortisol and mortality have been described in critical illness and post-cardiac arrest settings [17], suggesting that both adrenal insufficiency and excess may be maladaptive. In our study, persistent elevation appears more prognostically relevant, underscoring hypercortisolemia as a biomarker of failed stress resolution and disease severity in CS. This raises the question of whether glucocorticoid modulation offers therapeutic benefit in selected patients. In septic shock, hydrocortisone therapy remains contentious. The CORTICUS trial showed no mortality reduction, even among corticotropin non-responders, although vasopressor weaning was accelerated [20]. Six patients, that were excluded from further analyses in our cohort, received hydrocortisone with a mortality of 16.7%, compared to 44% in the overall population. This observation suggests that tailored corticosteroid therapy may benefit patients with relative adrenal dysfunction or refractory shock. However, no ACTH testing was performed, and mechanistic conclusions must remain cautious. Current trials try to address low dose corticosteroid therapy for cardiogenic shock patients [16]. Notably, cortisol and glucose trajectories in non-survivors were closely aligned, hinting at a shared, dysregulated stress axis.

Indeed, admission hyperglycemia was similarly pronounced, with a mean glucose level of 12,9 mmol/L—more than twice the upper limit of normal. Only four patients were normoglycemic at presentation, and although glucose levels declined over time, euglycemia was not achieved by day four. No significant difference was observed in glucose kinetics between survivors and non-survivors. Notably, patients without a prior diagnosis of diabetes exhibited higher mortality, indicating that acute stress-induced hyperglycemia—rather than pre-existing glycemic status—may be the principal driver of risk in this context.

This observation aligns with previous studies. Fefer et al. reported increased ICU morbidity in diabetic patients with acute MI, including infections and thromboembolic events [8], yet diabetes per se did not predict mortality in our cohort. Whitcomb et al. found that hyperglycemia was prognostic only in non-diabetic ICU patients [26], while Capes et al. showed that in acute MI, stress hyperglycemia conferred a 3.9-fold increased risk of death in non-diabetics, but only a 1.7-fold increase in diabetics [6]. Similarly, Marik and Goyal identified stress hyperglycemia as an independent mortality predictor in critical illness, regardless of diabetes status [10, 15].

Persistent hypercortisolemia likely drives this hyperglycemia through increased gluconeogenesis and insulin resistance [21]. Elevated cortisol levels have been associated with worse outcomes and higher glucose in acute coronary syndromes and CS [13]. In the SMART RESCUE trial, admission glucose predicted mortality in CS, particularly among non-diabetics [7]. The CardShock study further confirmed that severe hyperglycemia (≥ 16.0 mmol/L) was an independent predictor of in-hospital mortality, and was associated with systemic hypoperfusion markers including leukocytosis, elevated lactate, and acidosis [13]. Tian et al. also reported a U-shaped relationship between the stress hyperglycemia ratio and ICU mortality in CS, reinforcing the need for tailored glucose targets [22].

These findings carry important clinical implications. Intensive insulin therapy has shown mortality benefit in surgical ICU settings, as first demonstrated by Van den Berghe et al., who targeted glucose < 6.1 mmol/L(Van den [25]). Insulin’s cardioprotective properties include anti-inflammatory and vasodilatory effects [14]. However, the risk of hypoglycemia is substantial: 11.8% in the intensive group versus 1.8% in the standard care group in follow-up trials (Van den [24]). In our cohort, paradoxically, patients with initial glucose < 6.1 mmol/L had the worst outcomes, potentially reflecting abrupt glycemic shifts after prehospital insulin administration. This supports Van den Berghe’s proposal that insulin strategies should be individualized based on premorbid glycemic exposure (Van den [25]).

**Fig. 8:**
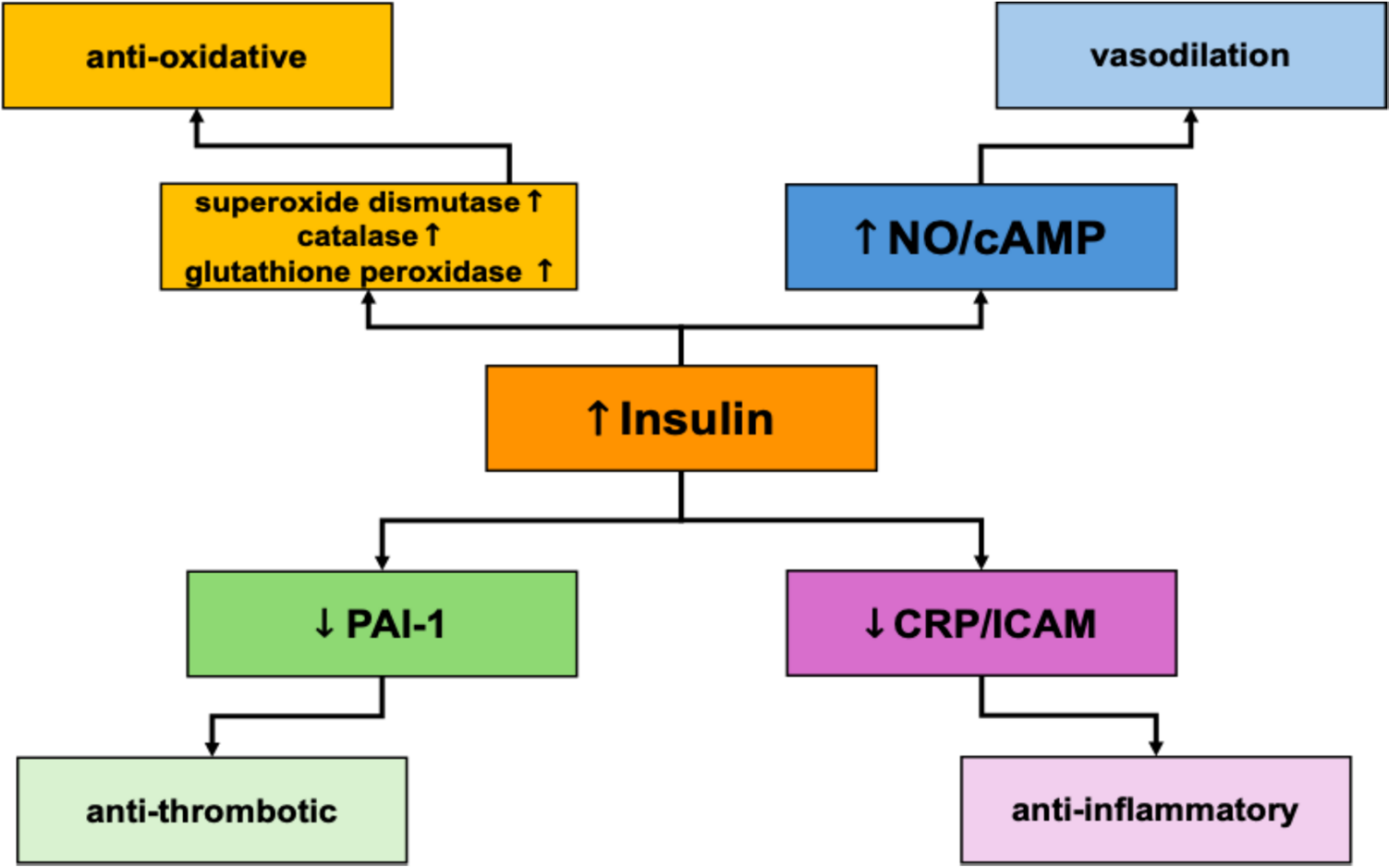
Cardiovascular effects of insulin in critical illness. Insulin exerts multiple cardioprotective actions, including attenuation of inflammatory responses, enhancement of endothelial function, inhibition of oxidative stress, and improvement of myocardial glucose uptake. These effects collectively support cardiac output and hemodynamic stability in acute settings such as cardiogenic shock

The lack of consistent mortality benefit and excess hypoglycemia in the Brunkhorst trial on intensified insulin therapy in severe sepsis led to early termination, emphasizing the dangers of overly aggressive glucose lowering [5]. Moving forward, insulin protocols in CS may need to favor moderate correction with real-time monitoring rather than tight control, particularly in hemodynamically unstable patients. In our study, all patients were treated with a standardized intravenous insulin infusion protocol targeting 140–180 mg/dL, ensuring comparability across participants and minimizing treatment-related heterogeneity [23]. Taken together, our findings (see Fig. 8 for an overview of insulin’s cardiovascular effects) identify admission cortisol and glucose levels as robust, rapidly available biomarkers for early risk stratification in infarction-related cardiogenic shock. The endocrine–metabolic response appears tightly coupled, and persistent dysregulation—manifested by sustained hypercortisolemia and stress hyperglycemia—portends poor outcome.

These parameters are measurable in routine clinical practice and may aid in triaging patients for closer hemodynamic monitoring or early therapeutic interventions. While current evidence does not support routine corticosteroids or intensive insulin therapy in CS, our data provide a compelling rationale for individualized endocrine profiling. Future trials should evaluate whether targeted modulation of the HPA axis and glucose metabolism, using real-time cortisol and glucose kinetics, can improve survival while minimizing adverse effects. Advances in continuous monitoring, AI-driven insulin dosing, and safer glucocorticoid thresholds may help translate these insights into clinical benefit [4].

While we examined cortisol kinetics by age and sex, these subgroup analyses are limited by small numbers and should be viewed as hypothesis-generating. The strong association of persistent hypercortisolemia with mortality is clinically relevant, given that some intensivists already use hydrocortisone as a catecholamine-sparing strategy in cardiogenic shock. Our findings therefore underscore the need for adequately powered trials to determine whether modulation of the hypothalamic–pituitary–adrenal axis can improve outcomes in this setting.

From a clinical perspective, the ability to measure cortisol and glucose at the bedside provides intensivists with rapidly available, low-cost biomarkers that can complement hemodynamic parameters in the early hours of shock. Translationally, integrating these endocrine–metabolic signals with established severity scores could refine risk stratification, support individualized therapy decisions, and identify patients most likely to benefit from novel interventions.

This study provides novel evidence that the trajectory—not merely the magnitude—of cortisol and glucose levels in cardiogenic shock holds key prognostic value. Integration of endocrine and metabolic profiling may define a new frontier in risk-adapted, physiology-guided management of this high-mortality condition.

### Strengths and Limitations

A major strength of this study is its prospective design with detailed temporal profiling of both cortisol and glucose in patients with infarction-related cardiogenic shock, a critically ill yet under-investigated population. Serial measurements over 96 h enabled assessment of dynamic trajectories rather than isolated values, and use of a standardized hospital laboratory enhanced analytical consistency. We further tested the robustness of our results by sensitivity analyses excluding patients who received corticosteroids, one predefined cortisol outlier, and those with suspected infection; findings remained materially unchanged, supporting the validity of our observations. The combined analysis of endocrine and metabolic markers provides novel insight into the stress response and its prognostic implications.

Several limitations should also be noted. This was a single-center study with a modest sample size, limiting generalizability. Dynamic adrenal testing (e.g., ACTH stimulation) was not performed, precluding definitive assessment of relative adrenal insufficiency. Because patients with acute infection or corticosteroid therapy were excluded, infection-related or iatrogenic hypercortisolemia is unlikely to have influenced the results. Finally, the observational design does not permit causal inference.

## Conclusion

Infarction-related cardiogenic shock elicits a profound metabolic stress response, with cortisol levels rising nearly fivefold above normal and accompanied by parallel increases in glucose. Both persistent hypercortisolemia and early hyperglycemia were independently associated with higher in-hospital mortality, whereas survivors demonstrated a more rapid normalization of these axes. These findings identify admission cortisol and initial glucose not only as markers of illness severity but also as translationally relevant, potentially modifiable targets. In particular, rapid glucose adjustment within the first 24 h may represent a novel strategy warranting evaluation. Early endocrine profiling could thus provide a clinically accessible tool for timely risk stratification and guide future interventional studies in cardiogenic shock.

## Supplementary Information

Below is the link to the electronic supplementary material.

Supplementary file 1 (PDF 299 KB)

## Data Availability

The data supporting the findings of this study are available from the corresponding author upon reasonable request.
