# Supplementary material for: The Prognostic Role of Cortisol and Glucose Dynamics in Cardiogenic Shock-Insights from a Prospective Observational Cohort
Source: J Cardiovasc Transl Res. 2025 Oct 18;18(6):1932–45. doi: 10.1007/s12265-025-10704-0 (PMC12700995; doi:10.1007/s12265-025-10704-0)

Supplement

Figure 1. Severity scores and their association with cortisol and glucose. Panel A: Sequential Organ Failure Assessment (SOFA) trajectories over 96 hours in survivors (blue) and non-survivors (green). Survivors showed a steady decline from median 8 to 3, whereas non-survivors remained elevated or worsened (12→14; group×time interaction $p<0.001$).

Panel B: Correlation coefficients of admission cortisol and glucose with severity indices. Cortisol correlated moderately with SOFA ($r=0.42$, $p=0.01$) and APACHE II ($r=0.39$, $p=0.02$), whereas glucose showed weak, non-significant associations (SOFA: $r=0.18$, $p=0.27$; APACHE II: $r=0.15$, $p=0.32$).

Panel C: Scatterplot of admission cortisol versus APACHE II scores demonstrating a positive association between higher cortisol concentrations and greater illness severity.

Panel D: Scatterplot of admission glucose versus APACHE II scores, illustrating only a weak and non-significant relationship.

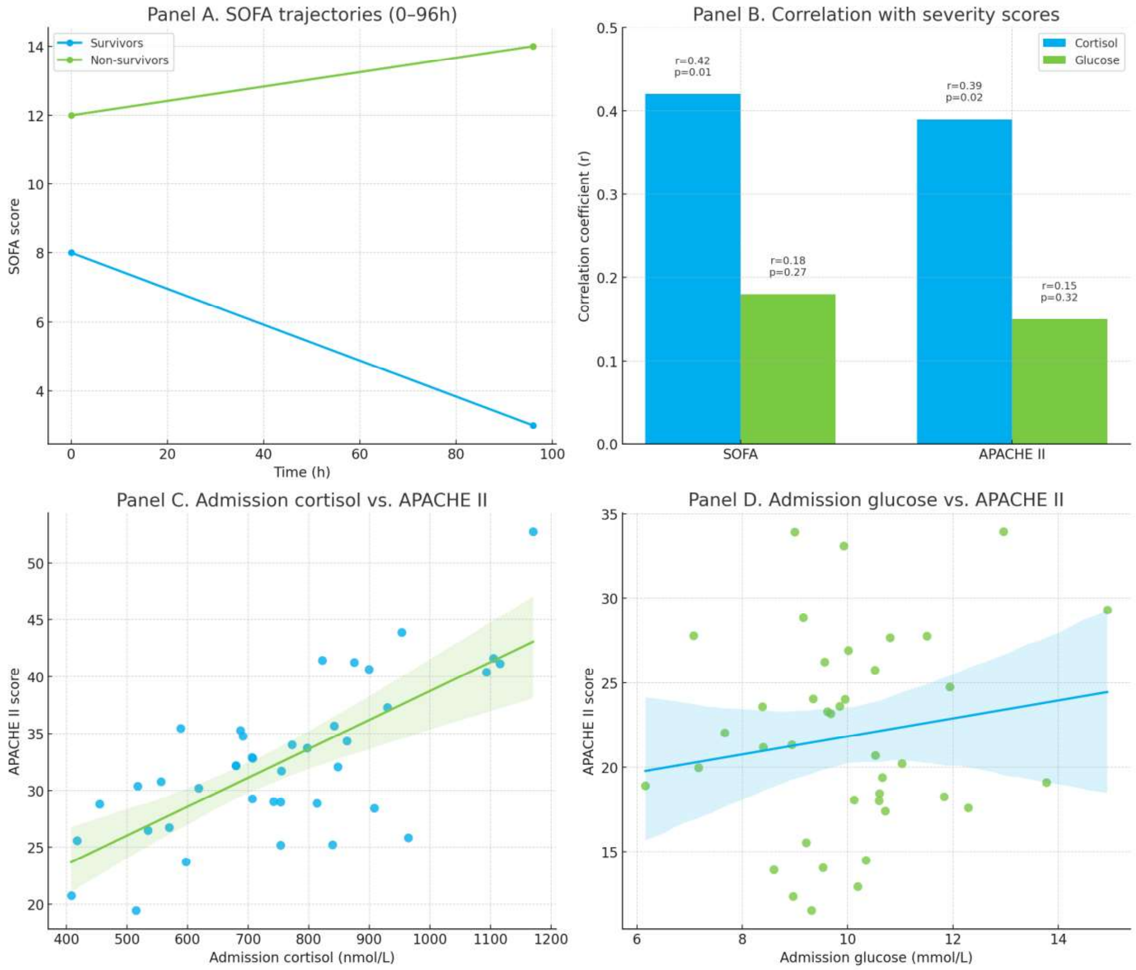

Supplement: Supplementary file 1 — (PDF 299 KB) [file 12265_2025_10704_MOESM1_ESM.pdf]
